# Mind the Interface Gap: Exposing Hidden Interface Defects at the Epitaxial Heterostructure between CuO and Cu_2_O

**DOI:** 10.1021/acsami.2c16889

**Published:** 2022-12-08

**Authors:** Aleksandar Živković, Giuseppe Mallia, Helen E. King, Nora H. de Leeuw, Nicholas M. Harrison

**Affiliations:** † Department of Earth Sciences, Utrecht University, Princetonlaan 8a, 3584CBUtrecht, The Netherlands; ‡ Department of Chemistry, Imperial College London, White City Campus, 80 Wood Lane, LondonW12 0BZ, United Kingdom

**Keywords:** heterostructure, CuO, Cu_2_O, density functional theory, band alignment, epitaxial interface

## Abstract

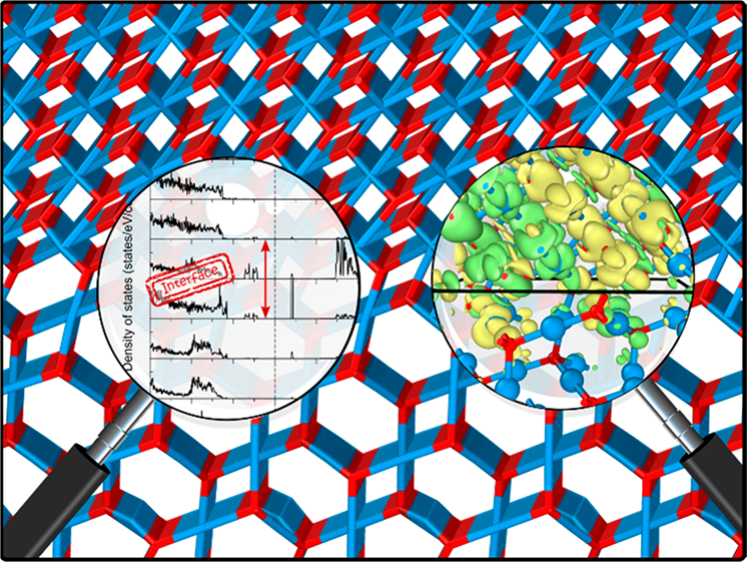

Well designed and optimized epitaxial heterostructures lie at the foundation of materials development for photovoltaic, photocatalytic, and photoelectrochemistry applications. Heterostructure materials offer tunable control over charge separation and transport at the same time preventing recombination of photogenerated excitations at the interface. Thus, it is of paramount importance that a detailed understanding is developed as the basis for further optimization strategies and design. Oxides of copper are nontoxic, low cost, abundant materials with a straightforward and stable manufacturing process. However, in individual applications, they suffer from inefficient charge transport of photogenerated carriers. Hence, in this work, we investigate the role of the interface between epitaxially aligned CuO and Cu_2_O to explore the potential benefits of such an architecture for more efficient electron and hole transfer. The CuO/Cu_2_O heterojunction nature, stability, bonding mechanism, interface dipole, electronic structure, and band bending were rationalized using hybrid density functional theory calculations. New electronic states are identified at the interface itself, which are originating neither from lattice mismatch nor strained Cu–O bonds. They form as a result of a change in coordination environment of CuO surface Cu^2+^ cations and an electron transfer across the interface Cu^1+^–O bond. The first process creates occupied defect-like electronic states above the valence band, while the second leaves hole states below the conduction band. These are constitutional to the interface and are highly likely to contribute to recombination effects competing with the improved charged separation from the suitable band bending and alignment and thus would limit the expected output photocurrent and photovoltage. Finally, a favorable effect of interstitial oxygen defects has been shown to allow for band gap tunability at the interface but only to the point of the integral geometrical contact limit of the heterostructure itself.

## Introduction

Material interfaces play an important role for a wide spectrum of technologies, such as semiconductors, spintronics, or quantum devices, to name a few. They span over a spectrum of properties and processes ranging from transport and confinement of electrical charge, mechanical strain, and accumulation of defects and impurities to thermal barrier effects and atomic reconstructions.^1−6^ Interfaces possess an increased degree of complexity and disorder when compared to the bulk and are intrinsically much harder to characterize experimentally.^7^ Thus, understanding the way distinct systems interact when grown or deposited together is of vital importance to achieve a high-quality interface with controlled structure and tunable properties.

One field where interfaces are of paramount importance is renewable energies, out of which solar energy is by far the most abundant.^8^ The conversion of energy from sunlight into electrical energy or chemical fuels will be vital in sustainable development due to its large capacity, cleanliness, and low environmental and economic cost.^9^ To meet such demands, the materials forming the interfaces need to be abundant, scalable, and compatible with low-cost fabrication processes.

Cuprous (Cu_2_O) and cupric (CuO) oxide are among the candidates that meet many of these necessary requirements while offering potentially promising performance in terms of photocurrent and photovoltage output.^10,11^ They are also gaining increased attention in the field of battery electrodes,^12^ photonic crystals,^13^ hydrogen production,^11^ water treatment,^14^ and even antibacterial activity.^15^ However, low absorption and poor chemical stability in aqueous conditions combined with fast electron–hole recombination hinder the onset of photocurrent and limit the practical utilization of Cu_2_O or CuO.^16,17^ For example, in order to efficiently absorb sunlight, Cu_2_O films must typically be at least 1 μm thick. However, the minority carrier (electron) diffusion length is limited to 20–200 nm depending on the synthesis process, resulting in inefficient collection of photogenerated carriers.^18^ Similarly, CuO suffers from low electronic conductivity^19^ and low mobility of charge carriers.^20^ Therefore, it is evident that efficient separation of the photoexcited electron–hole pairs, before they are able to recombine, is crucial.^21^

Constructing a suitable interface between Cu_2_O and CuO is expected to suppress some of the losses as a result of spatial separated electron–hole carriers at the interface, thus inhibiting recombination; i.e., the conduction band minimum (CBM) of Cu_2_O is postulated to be slightly higher than the CBM of CuO and the valence band minimum (VBM) of CuO lower than the VBM of Cu_2_O. On top of that, CuO is expected to also serve as a protective layer against Cu_2_O photocorrosion.^16^ Initial experimental findings have observed an increased photocurrent density, improved electron–hole separation, and charge transfer efficiency due to an anticipated enhanced transfer of photogenerated electrons from Cu_2_O to the conduction band (CB) of CuO and holes from CuO to the valence band (VB) of Cu_2_O at the Cu_2_O/CuO heterojunction.^21−24^

Cu_2_O/CuO heterojunction structures can be synthesized in a variety of ways. Bao et al. grew a CuO overlayer on top of two distinct Cu_2_O nanocrystals (grown to expose the (111) and (100) facets) using solution-phase methods and observed catalytic CO oxidation pathways that depended strongly on the exposed crystal planes.^25^ Baek et al. produced aligned Cu_2_O/CuO and Cu_2_O:Sb/Cu_2_O/CuO heterostructures by oxidizing Cu_2_O under reduced oxygen partial pressure.^26^ They measured an improved photocurrent density and onset potential, when compared to pristine Cu_2_O, and interpreted it to originate from the enhanced charge transport efficiency owing to less interface defects and a “decent conduction band offset”. However, growing a CuO overlayer of a thickness greater than 40–50 nm produced porous grains which crack the interface presumably due to internal stress. Thus, the interface overlayer of CuO should be fabricated at an optimum thickness and high crystallinity.

As briefly outlined above, CuO/Cu_2_O composites are speculated to have potential application in spintronic devices as well. This stems from magnetic measurements indicating interface-based room temperature ferromagnetism in bulk^27^ as well as microsphere^28^ CuO/Cu_2_O compounds. The ferromagnetism was traced back to oxygen vacancies present at the interface which introduce one localized orbital with two unpaired electrons via which the doped electrons can hop between different Cu ions connecting them ferromagnetically (double-exchange). Heterostructures of CuO and Cu_2_O have been investigated for photocatalytic H_2_ evolution reactions on both nanomaterials and films.^29−31^ More recently, local photoluminescence properties of CuO/Cu_2_O bilayers were assessed.^32,33^ There, light emission intensity maps at photon energies of 1.5–3.3 eV were measured for samples annealed at different temperatures, yet a definite band assignment was not conclusive as the spatial resolution was limited to a few μm. Izaki et al. grew a layer of CuO on top of a Cu_2_O layer and analyzed the photovoltaic (PV) properties of the stacked layers.^34^ The heteroepitaxially grown layers were found to possess nanopores of several hundred nanometers, most likely arising from the large lattice mismatch of about 20%, which resulted in the disappearance of visible light emission and PV performance for both the CuO and Cu_2_O layers. Such results reiterate the need to retain high crystallinity, a small lattice mismatch, and low concentration of defects at any interface in order to achieve high efficiency PV performance. Izaki et al. further stacked Cu_2_O layers on electrodeposited CuO layers and treated the samples at various temperatures.^35^ A minimal strain between the layers and maximal PV performance was noticed for samples heated at 423 K, while heating at 473 K and above resulted in deteriorated bilayers and PV features disappearing entirely. Khoo et al. created electrodeposited and annealed directly stacked Cu_2_O/CuO layers and measured the conduction band offsets (CBO) to be between 0.80 and 0.96 eV, depending on the annealing temperature (523 and 673 K, respectively).^36^

In summary, despite the improved photostability and enhanced photocurrents the CuO/Cu_2_O interface displays, a high density of defects was noted to occur when the crystal distortion and strain at the interface are not precisely suppressed and controlled.^26^ The exact origin of the defects occurring at the interface is not entirely clear and is postulated to arise from the strained Cu–O bonds. Furthermore, the exact band alignment and accompanying electronic interface structure of Cu_2_O and CuO remain unknown (e.g., whether the CBM of CuO is higher^37^ or lower^26^ than the CBM of Cu_2_O) and are limited to rather speculative schematic illustrations or simplistic based on measurements performed on separate materials.^21^ Relevant density functional theory (DFT) works are scarce and often found to be limited to nanostructures^38^ or interfaces with metallic copper,^39^ yet a thorough theoretical investigation of this interface system had yet to be reported.

In this work we investigate the CuO/Cu_2_O heterostructure and analyze its interfacial geometry, band alignment, as well as charge, spin, and orbital degrees of freedom present at the interface; something which is very intricate or inaccessible in current experimental efforts.^40^ To do so, a range of simulations are performed, where first the suitable surface terminations and mutual alignments are analyzed, upon which the degree of epitaxial strain is evaluated, and finally, coherent interface structures are created. Subsequently, the junction structure is treated using hybrid density functional theory (where nonlocal Fock exchange is known to remedy some of self-interaction and delocalization of semilocal functionals^41^) and a detailed analysis of the underlying atomic structure, electronic structure, and spin density performed. An attempt is made to answer some of the following questions: (i) What is the effect of strain and surface chemistry on band edge positions of individual Cu_2_O and CuO surface planes when compared to the aligned epitaxial heterostructure? (ii) To what extent does the band interface diagram deduced from the properties of constituent materials deviate from an explicitly modeled CuO/Cu_2_O epitaxy?

## Computational Details

Calculations were performed using density functional theory methods applying a linear combination of atomic orbitals basis set as implemented in the CRYSTAL17 software.^42,43^ Atoms were described using literature available basis set with minimal modifications: copper^44^ and oxygen.^45^ One additional *d*-function (with an exponent of 0.172 bohr^–2^) was added to the Cu basis set, while the remaining basis sets were taken without further modification. The B3LYP hybrid functional was used throughout the work, unless stated otherwise.^46,47^ The mixing of nonlocal Fock and semilocal exchange provides a reliable representation of electronic and structural properties of a range of oxide compounds.^41,48,49^ Long range dispersion corrections were included using the semiempirical D3 approach of Grimme et al. with Becke–Johnson damping.^50−52^ The Coulomb and exchange series are summed directly and truncated using overlap criteria with thresholds of 10^–8^, 10^–8^, 10^–8^, 10^–8^, and 10^–16^ as described previously.^42,53^ The condition for the self-consistent field (SCF) convergence was set to 10^–6^ au on the total energy difference per cell between two subsequent cycles. Reciprocal space was sampled according to a regular sublattice with a shrinking factor (input IS) of 11 for bulk Cu_2_O and 9 for the (111) surface of Cu_2_O. Since the unit cell of cupric oxide is highly anisotropic, shrinking factors of (5 13 5) were used for bulk CuO, (5 3) for the (012)_mag_ and (210) _mag_ surfaces, (5 5) for the (111) _mag_ surface, and (13 7) for the (00–4) _mag_ surface of CuO, maintaining a k-point spacing of approximately 0.05 Å^–1^.

Coherent (epitaxially constrained) interface structures were produced using pymatgen.^54−56^ Pymatgen implements the Zur and McGill lattice matching algorithm^57^ which generates possible domain-matches superlattices between a film and a substrate for a given set of area, lattice vector angle, and lattice vector length mismatch criteria. However, it does not (at present) take into account the complex chemistry of the interface. Nevertheless, it serves as a good starting point to reduce the number of possible structures by focusing on systems that build an epitaxial repeat unit within a specific tolerance.

The surfaces (and interfaces) were modeled as two-dimensional periodic slabs, where no three-dimensional periodicity was imposed. To characterize the surface, the surface energy (γ) as a measure of the thermodynamic stability has been calculated through the following expression:
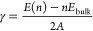
1where *E*(*n*) is the energy of the slab containing *n*-layers, *E*_bulk_ the energy of the bulk, and *A* the area of one side of the slab.

The band alignment based on individual (noninteracting) compounds was determined using the ionization potential (IP) and electron affinity (EA) energies for which the vacuum level served as a common reference. Two definitions for the IP were considered: (i) bulk-based IP, where the IP is defined as^4,58^
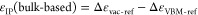
2where Δε_vac-ref_ is the difference between the electrostatic potential at large distance from the slab (which in our case is zero) and the bulk-like reference level in the slab (the 1s states of Cu in the middle of the slabs) while Δε_VBM-ref_ is the difference in eigenvalue energy between the VBM and reference level from bulk calculations, and (ii) surface-sensitive IP, where the IP is taken as the difference between the vacuum level and the highest occupied level in the slab model. In both cases, the electron affinity is obtained by subtracting the obtained band gap value from the ionization potential.

The specific adhesion energy, a measure of the energy gained once the interface boundary between two surfaces (s1 and s2) is formed, is given by
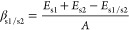
3where *E*_s1_ and *E*_s2_ are total energies of the respective slabs and *E*_*s*1/s2_ is the final interface energy.

The specific interface energy, defined as the excess energy resulting from the energy balance described by the Dupré’s relation,^59^ is given by
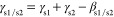
4where γ_s1_ and γ_s2_ are surface energies of the respective slabs forming the interface, and β_*s*1/s2_ is the adhesion energy defined earlier.

Using predicted surface energies, the equilibrium morphology of CuO nanocrystals has been constructed according to the Wulff theorem.^60^ Graphical visualizations have been made using OVITO.^61^ The charge density difference was defined as^62,63^ Δρ = ρ_s1/s2_ – (ρ_s1_ + ρ_s2_), where ρ_s1_ and ρ_s2_ are densities of independent surfaces and ρ_s1/s2_ is the electron density of the interface.

## Results and Discussion

### The Bulk

Cu_2_O is characterized by a high symmetry cubic structure and a unit cell containing four Cu atoms forming a tetrahedron around each of the two O atoms. The calculated lattice parameter, using the global hybrid B3LYP functional, is 4.26 Å, which is in excellent agreement with measured X-ray diffraction values of 4.27 Å.^64^ Overall, regardless of whether they are global or range-separated, hybrid functionals perform very well when it comes to reproducing the geometry of cuprous oxide. For a full overview of tested functionals, see Table S1. The Kohn–Sham (KS) single-particle electronic band gap is computed at 2.22 eV with B3LYP and located at the Γ-point reproducing the experimentally measured direct nature transport gap of 2.17 eV in good agreement.^65,66^

CuO crystallizes in a lower symmetry monoclinic structure with Cu atoms organized in a square planar coordination arrangement with four surrounding O atoms, while each of the O atoms is located in the center of a distorted Cu tetrahedron.^67^ The measured lattice parameters and angles are *a* = 4.72 Å, *b* = 3.40 Å, *c* = 5.04 Å, and β = 99.5°.^20^ As a result of the nominally Cu^2+^ d^9^ electronic configuration, there are unpaired spins present in the system giving rise to intrinsic magnetism. CuO orders antiferromagnetically below 225 K, with an intrinsic magnetic moment of 0.68 μB present on the Cu atoms and a propagation vector along the (1/2 0 −1/2) direction.^68,69^ This experimentally observed ordering cannot be reproduced in the standard conventional unit cell but rather in a doubled cell (labeled as magnetic in this work) containing 16 atoms. The lattice vectors of the magnetic cell are obtained by **a′** = **a** + **c**, **b′** = **b**, and **c′** = −**a** + **c**, where **a**, **b**, and **c** are lattice vectors of the conventional unit cell of CuO.^70^ For a variety of hybrid functionals tested here, the lattice parameters are reproduced well, up to a few percent from experimental measurements (for complete results, see Table S2). However, the same does not hold for the electronic structure, at least at first sight. The low temperature experimental gap is usually reported in to be in the range of 1.3–1.5 eV,^71^ while the calculated values overestimate the gap substantially. The band gap of CuO had a Mott–Hubbard character, which presents fundamental problems for reproduction within the KS eigenvalue scheme.^72^ Local and semilocal functionals predict a metallic ground state of CuO, which is a well-documented shortcoming of these approximations.^73^ Global hybrid functionals like B3LYP or PBE0, regardless of the basis set approximation, reproduce the correct nature of the electronic ground state but yield band gap values around 3 and 4 eV, respectively, which is more than double the experimental value. A screened Coulomb hybrid functional like HSE06 generates a gap of about 3 eV for CuO, while middle-range corrected (HISS) and long-range corrected hybrid functionals (CAM-B3LYP or LC-ωPBE) result in gaps larger than 4 eV (full results available in Table S3).

Not only is the value of the band gap of CuO significantly overestimated with respect to experimental measurements, but it is also much larger compared to the gap of Cu_2_O, which is a potentially serious issue when constructing the band alignment between the two compounds as one could misinterpret the type and offsets of the interface entirely. Leaving no stone unturned, we tuned the portion of Fock exchange entering global hybrid functionals as well as the separation length (ω) in the range-separated HSE06 functional, in addition to the various basis set and functional combinations tested earlier (Figure S1). There was no crossing point observed that would reproduce the correct experimental ordering of *E*_g_ (Cu_2_O) > *E*_g_ (CuO) and at the same time reproduce the correct physics and chemistry of these two systems.

To go beyond the electronic band gap evaluation based on one-electron eigenvalues from ground state DFT, the fundamental energy gap *G* was estimated from total energy differences: *G*_SCF_ = IP(*N*) – EA(*N*) = *E*_SCF_(*N* – 1) + *E*_SCF_(*N* + 1) – 2*E*(*N*), where *N* is the ground state number of electrons in the system and *E* the total energy, IP(*N*) the ionization energy, and EA(*N*) electron affinity of the system.^74^ Orbital relaxation effects were allowed, yielding self-consistent *E*_SCF_(*N* ± 1) values. Since the electrostatic energy of a charged supercell calculations diverges, one must include a compensating homogeneous background charge.^75,76^ No further corrections have been used, unlike when needed when computing defect formation energies,^77−80^ as the contributions of the *N* + 1 and *N* – 1 systems cancel out in *G*_SCF_. The calculated fundamental gaps of cuprous oxide and cupric oxide are *G*_SCF_(Cu_2_O) = 2.33 eV and *G*_SCF_(CuO) = 2.88 eV, which agree well with the eigenvalue gaps of 2.22 and 3.02 eV, respectively, confirming the soundness of the earlier obtained KS values (for full results with respect to supercell size, see Table S4).

It is worth noting at this stage that both the KS eigenvalue and total energy fundamental gaps were computed on the perfect ground state zero-temperature antiferromagnetic (AFM) collinear spin ordering (which is often in literature labeled as AFz^81,82^). Imposing any different spin arrangement results in a state that is higher in energy than the AFz one. But the electronic structure of CuO is found to undergo significant changes when the spin configuration is changed. For example, inducing 5% spin alterations (in a supercell with each lattice dimension larger than 10 Å) reduces the gap from 3.02 to 2.62 eV for an energetic cost of about 110 meV/spin-flip (see Figure S2). These values are in accordance with the strongest spin exchange coupling value determined for CuO in the commensurate phase (around 100 meV, depending on the functional or prepared sample^83^) which favors the observed antiferromagnetic alignment. As the temperature increases up to 213–215 K, the 3D AFM long-range spin order gets progressively destroyed and transforms into a helicoidal incommensurate AFM structure up to approximately 230 K and then further to an incommensurate sinusoidal collinear structure with half of the spins aligned to finally become paramagnetic above 230 K.^84,85^ A detailed electronic structure across these spin and temperature regions is, to the best knowledge of the authors, presently unavailable, but it does suggest that the band gap would alter drastically as a result of spin flips or spin–lattice relaxations induced by the destruction or locking of a particular magnetic state (such as recently discovered in NiO and MnF_2_ responsible for their modified optical response^86^).

While the excitonic series of Cu_2_O is well resolved^87,88^ and the fundamental band-to-band transition measured around 2.1 eV regardless of the experimental synthesis and measurement setup,^89−92^ high-quality spectral or optical data for CuO are scarce while those that exist have a range of band gap values from 1.0 to 2.1 eV ^93^ or more recently even outside the visible range, as discussed further below. This makes a sensible comparison between the theoretical values computed for cupric oxide and those measured experimentally extremely challenging and imprecise. Given the computed sensitivity of the band structure to the spin arrangement and the neglected intrinsic defects, electron–phonon, and spin–lattice relaxations across the Néel and higher temperatures, the computed values can be seen as the upper boundary limit for a perfect cupric oxide in the low temperature regime. With this solid foundation of the bulk we are motivated to further explore the surface structures of both copper oxides and their interfaces.

### Surface Behavior

As it is exceedingly difficult to establish the atomistic composition and structure of a buried interface experimentally, it is necessary to posit a thermodynamically reasonable interface from general considerations. This is greatly facilitated by a detailed analysis of the structure and energetics of the component surfaces. Svintsitskiy and co-workers^94^ analyzed the initial steps of CuO nanopowder reduction and observed the formation of (111)Cu_2_O|(−111)CuO epitaxy as a result of small deviations in interplanar spacing and the similar arrangements of copper atoms in the outlined crystal faces. Further, Zhu et al. characterized microstructure of CuO–Cu_2_O and observed the dominant planes to be the (111) of Cu_2_O and (111) and (−111) of CuO.^24^ This motivated the choice of these surfaces for further epitaxial processing and analysis.

Starting from B3LYP relaxed geometries, the Cu_2_O bulk was cut along the (111) crystallographic plane. We further considered only stoichiometric nonpolar surfaces to avoid complex reconstructions and stabilization issues. The B3LYP relaxed surface energy of the Cu_2_O(111) slab stabilized quickly with an increasing number of layers and reached a value of 0.95 J/m^2^, in good agreement with earlier works.^95,96^ The electronic structure of the slab is found fully converged for thickness of 11 Å to a direct band gap of about 2.25 eV, adding no noticeable surface states to the bulk band structure.

When cleaving the CuO bulk, the notation of the Miller indices is altered as we start from a magnetic rather than the conventional crystallographic cell. The (−111)_conv_ plane in the conventional cell corresponds to the (012)_mag_ plane in the magnetic cell, as a result of the lattice transformations outlined earlier. This allowed us to start from the bulk ground state AFz magnetic ordering and propagate it correctly onto the surface.

The relaxed surface energy of CuO(012)_mag_ is calculated at 0.94 J/m^2^ and corresponds well to literature values, e.g., 0.89 J/m^2^ obtained using DFT+U calculations.^97^ In the final slab electronic structure, new states localized at the surface are found inside the otherwise pristine bulk electronic structure, reducing the overall band gap by almost 1 eV (for full convergence tests see Figure S3). This is evidenced by a large number of newly formed dangling bonds which originate from 3-fold coordinatively unsaturated Cu and O surface atoms (Figure S4). Consequentially, the oxidation state is lowered and the magnetic moment of the copper atoms reduced as well. This goes well in hand with experimental measurements, where a mixture of Cu^1+^ and Cu^2+^ valence states has been noted to exist at the surface of CuO nanoparticles.^98^ The analysis of successive surfaces of CuO found similar trends (see Table 1), where all of the slab electronic structures had extra localized states in the pristine bulk band gap.

**Table 1:** Relaxed Surface Energies and Band Gaps of CuO Surfaces under Scrutiny^a^

		B3LYP		HSE06	
Miller index		surface energy (J/m^2^)	band gap (eV)	surface energy (J/m^2^)	band gap (eV)
(−1 1 1)_conv_	(0 1 2)_mag_	0.935	2.04	1.259	1.97
(1 1 1)_conv_	(2 1 0)_mag_	0.863	2.46	0.982	2.41
(0 1 1)_conv_	(1 1 1)_mag_	0.890	2.65	1.157	2.62
(2 0 −2)_conv_	(0 0 −4)_mag_	1.329	2.23	1.383	1.95
		bulk	3.02	bulk	3.19

a Values were obtained using two hybrid functional approximations. Complete corresponding notation of low surface Miller indices between the conventional and magnetic cell is listed as well.

Furthermore, these surface electronic structure results are obtained for a ground-state low temperature unperturbed collinear spin arrangement carefully propagated from the bulk onto the surface. Simulating spin flips on individual surface atoms reveals further band gap alterations at a very low energetic cost. For example, the band gaps of the CuO (−111)_conv_ and (111)_conv_ surfaces change from 2.04 eV and 2.46 eV to 1.80 eV and 2.02 eV, respectively, for only 20–40 meV per spin. Such spin fluctuations are energetically even more favorable on the respective surfaces than in the bulk, yet the magnitude of the gap alteration remains consistent, with a maximal reduction of approximately 0.40 eV. Finally, results are also consistent between the B3LYP and HSE06 functionals.

**Figure 1:**
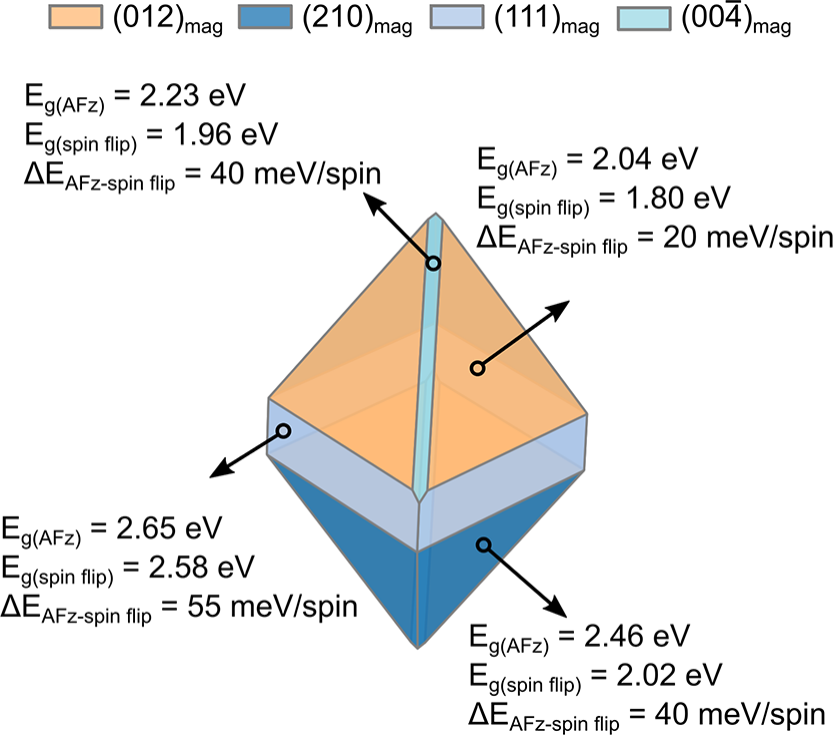
Wulff morphology of the pristine monoclinic CuO crystal with the corresponding electronic band gaps for an ideal magnetic ordering propagated from the bulk as well as a magnetic ordering where a small amount of surface spins is altered. Values were obtained using B3LYP.

The surface state influenced gaps present on each crystal facet are also indicated in the equilibrium morphology of CuO depicted in Figure 1. Given the sensitivity to the local spin coupling and the particular surface exposed, one concludes that the computed fundamental band gaps for CuO nanostructures are in the range 1.80–2.58 eV. This rather wide range may help to explain the discrepancies in measured data of CuO.

Using the predicted surface energies for the selected Miller indices, the equilibrium morphology of CuO nanoparticles is constructed and depicted in Figure 1. It is noted that the facets of CuO nanocrystals exhibit fundamentally different electronic structures, which taking into account spin alterations considered cover a range from 1.80 to 2.58 eV, spanning almost 1 eV. Despite the legitimate suspicion that these results are merely a product of the employed simulation parameters, they could serve to bridge the large range of experimentally noted values and disparities within.

For example, in a recent review on transparent conducting oxides, Spencer et al.^99^ gathered some of the more recent measurements on CuO samples where the band gap energy ranges from 1.35 to 2.03 eV, depending on the synthesis method and substrate used. Jhansi et al.^100^ created CuO thin films on a glass substrate and observed band gaps ranging from 1.8 to 2.9 eV, depending on the substrate temperature. Panda et al. fabricated thin films containing copper oxide phases and measured gaps of 2.45 and 2.25 eV for Cu_2_O and CuO, respectively.^101^ And finally, Tripathi et al.^102^ synthesized CuO nanostructures and treated them with KMnO_4_ to observe a change in the gap going from 1 eV to almost 4 eV, depending on concentration increase of KMnO_4_. Values spanning such a broad range imply not only the sensitivity of CuO toward the experimental setup but also large tunability of properties as samples with a gap below 1 eV would be entirely semiconducting and dark, while samples with a gap of 4 eV would be fully transparent. The origin of these variations has not been established in previous works. Our results indicate that such values could all be intrinsically part of the CuO nanosystem, where different synthesis methods and treatment temperatures expose distinct crystal planes in the morphology of the crystal and lock the system in a particular spin state, yielding distinct electronic and magnetic structures.

It is worth noting that such band gap reductions between the bulk and the corresponding crystal surfaces are not unique to cupric oxide. For example, Taylor et al. observed surface states that drastically alter the band gap of a range of orthorhombic perovskites, namely, CaSnO_3_, SrSnO_3_, BaSnO_3_, and SnTiO_3_,^103^ with some of the newly formed unoccupied states straddling the reaction levels for favorable water splitting applications. However, such effects in the electronic structure of CuO have so far been neglected.

**Figure 2:**
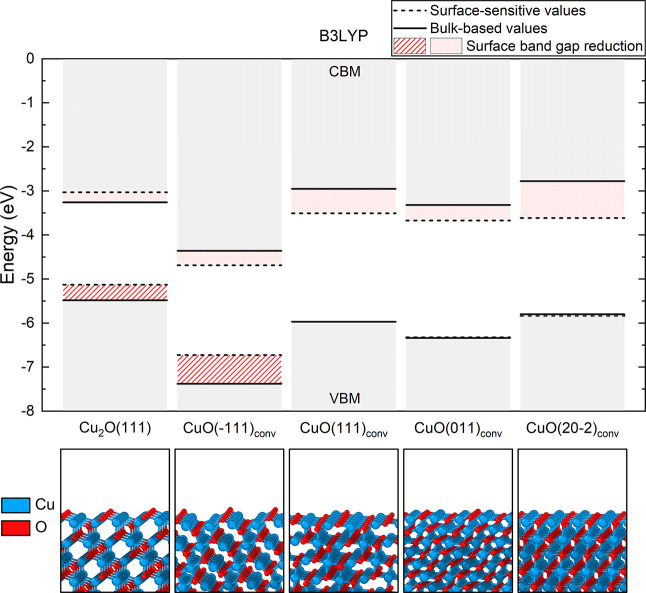
Band alignment based on individual compounds estimated from the ionization potential and electron affinity values obtained from surfaces of Cu_2_O and CuO together with their corresponding crystal structures. Solid lines denote the bulk band gap, while the dashed lines highlight their surface reduction. Energies are obtained using the B3LYP functional and aligned with respect to the vacuum.

An estimated band alignment scheme based on the individually computed compound values is presented in Figure 2. The creation of a surface does reduce the gap yet does not alter the ordering of the VBM and CBM between respective surfaces. However, the (−111)_conv_ cut of CuO does show considerably shifted values for the alignment with Cu_2_O compared to the other considered surfaces of CuO. There the VBM of Cu_2_O and CBM of CuO are separated by 1.13 eV (0.45 eV) for values taken from the bulk (surfaces), suggesting a Z-type-like scheme alignment. For the remaining CuO surfaces aligning with Cu_2_O, the CBM of CuO is positioned higher than the CBM of Cu_2_O while at the same time the VBM of CuO is positioned lower than the VBM of Cu_2_O, resulting in a straddling gap (from bulk values). The lowering of the gap for the (111)_conv_, (011)_conv_, and (20–2)_conv_ surfaces of CuO results in their CBM being placed lower than the CBM of Cu_2_O, yielding a staggered type of alignment.

The computed ionization potential (IP) and electron affinity (EA) of Cu_2_O and CuO compare reasonably well with experimental values. For Cu_2_O, the B3LYP computed EA and IP values are −3.25 eV and −5.48 eV (from a bulk-based definition), respectively, matching well the measurements of −3.20 eV (EA) and from −5.0 eV to −4.0 eV (IP).^17,104^ For CuO, the IP/EA measurements are somewhat scattered again, similar to the band gap values, with the IP ranging from −5.34 eV to −4.80 eV and the EA extending from −3.23 eV to −4.07 eV.^104^

### Interfaces between CuO and Cu_2_O

From the independent compound band alignment it is clear that a definite conclusion for the junction type and VB/CB offsets between Cu_2_O and CuO cannot be obtained. To gain further information about the interface structure and how it affects the band alignment, explicit interfaces have been simulated. We start by analyzing the lattice mismatch and strain between the two materials, where CuO (assuming a perfectly ordered magnetic ground state) is treated as a film on top of Cu_2_O, which is regarded as a substrate. Figure S5 depicts the results of the obtained configurations using a maximum search area criterion of 400 Å^2^ between various combinations of Cu_2_O and CuO slabs. Interfaces formed with a lattice strain which is larger than 5% have been neglected as they are unlikely to form experimentally.

From the performed analysis, two potential candidate interface structures have been identified: one reproducing the proposed alignment between the CuO(−111) and Cu_2_O(111) surfaces and one matching the CuO(20–2) and Cu_2_O(111) surfaces. However, in the latter, a suitable bonding mechanism connecting both materials could not be found, leaving the CuO(−111)_conv_/Cu_2_O(111) interface with the smallest lattice mismatch (slightly under 5%) and reasonable matching area that is at the same time computationally tractable for the employed DFT model.

**Figure 3:**
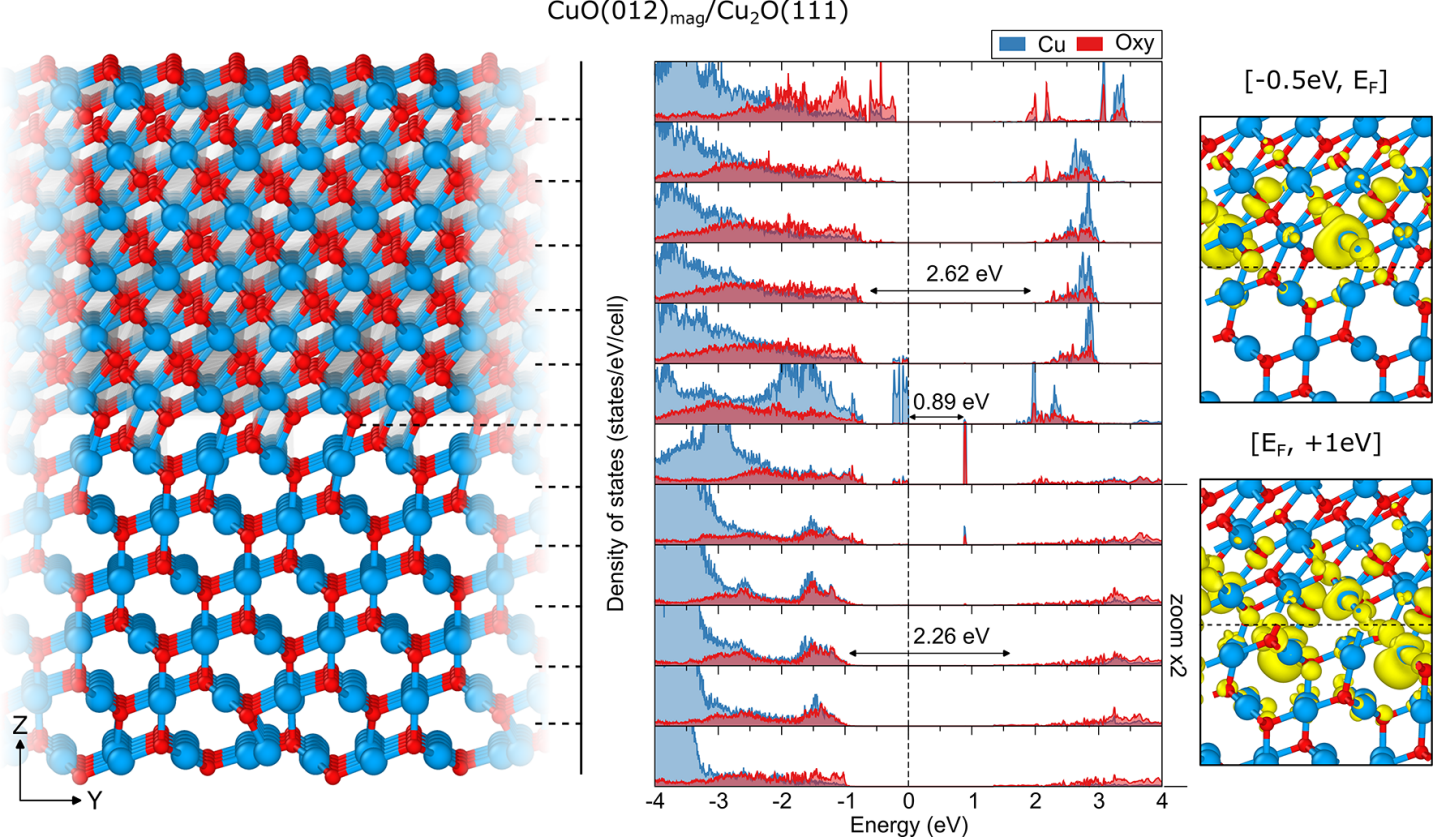
Relaxed atomic structure of the interface formed between the CuO(−111)_conv_ slab acting as a film and the Cu_2_O(111) slab acting as the substrate (left) together with the species-resolved layer-projected electronic densities of states (LPDOS, middle) and partial spin-resolved densities around the Fermi level (right). Dashed lines in the relaxed structure indicate the layers separation chosen for the LPDOS projections. Zero on the energy axis of LPDOS refers to the Fermi level of the interface. Isosurfaces are reported for values of 0.005 e/Å^3^ for VB and 0.0025 e/Å^3^ for CB states. Values were calculated using the B3LYP functional.

The atomically relaxed CuO(−111)_conv_/Cu_2_O(111) is shown in Figure 3. The outermost oxygen atoms present at both surfaces constitute the main bridging mechanism at the heterojunction contact. The main connecting points were identified to be the undercoordinated oxygen atoms from each surface with the undercoordinated copper atom at the opposite slab. Upon geometry relaxation, the undercoordinated CuO oxygen atoms formed a bond with the Cu_2_O copper atoms closest to the interface. There are two distinct bonds formed: the former with a previously 2-fold coordinated copper at a final bond length of 1.91 Å, inducing an electron transfer and altering the oxidation state of the Cu^1+^ into effectively Cu^2+^, with a final spin-only magnetic moment of almost 0.5 μ_B_. The latter bond is formed with a singly coordinated copper atom found at the Cu_2_O(111) surface, where the distance to the O is 1.82 Å, which is comparable to the usual 1.85 Å Cu–O bond length in the bulk of Cu_2_O. After relaxation, the two slabs are displaced (slide) with respect to one another, allowing the system to accommodate the Cu–O–Cu bond angle in the distorted tetrahedra around the interface oxygen that binds the newly formed Cu^2+^ cation.

The structural compatibility between the two oxide phases is further confirmed in the high adhesion energy of more than 2.1 J/m^2^ (Table 2). Consequently, a low interface energy of around 0.3 J/m^2^ is computed, indicating a small energetic gain when CuO is deposited as a film on top of a Cu_2_O substrate. In classical nucleation theory the barrier to nucleation arises due to the higher energy associated with a surface compared to bulk material and the strain that occurs across an interface due to the structural differences between two materials that are interacting.^105^ Thus, the high epitaxy between CuO and Cu_2_O would result in a lower strain between the two materials, expressed as a larger adhesion energy and smaller interface energy, which is in very good agreement with experimental observations. Moreover, the calculated adhesion energy of a Cu_2_O film epitaxially grown on a CuO substrate is almost 0.3 J/m^2^ lower when compared to the deposition of CuO on top of Cu_2_O, suggesting a more stable synthesis pathway of CuO when used as a film rather than a substrate.

**Table 2:** Adhesion and Interface Energies for Selected Interfaces between Cuprous and Cupric Oxide^a^

heterointerface system	interface dipole (D)	area (Å^2^)	thickness (nm)	adhesion energy (J/m^2^)	interface energy (J/m^2^)
CuO(−111)_conv_/Cu_2_O(111)	–4.44	62.89	2.4	2.12	0.29
	–4.85	62.89	2.9	2.12	0.35
Cu_2_O(111)/CuO(−111)_conv_	4.44	63.11	2.4	1.83	0.42

a Values obtained using B3LYP. Interface dipole moment given along non-periodic direction (Z).

A layer projected density of states (LPDOS) has been calculated for the CuO(−111)_conv_/Cu_2_O(111) heterojunction across the aligned materials, as shown in Figure 3. This way, changes in the PDOS can be monitored layer-by-layer, moving from the bulk-like region to the interface between the two materials. Starting from the substrate region, the band gap energy of Cu_2_O in the bulk-like region is calculated around 2.26 eV, corresponding well to the free-standing Cu_2_O(111) slab as well as bulk. However, the electronic structure changes drastically when approaching the interface. At a distance of 2–3 atomic layers from the actual interface between the two materials, new defect-like states have emerged, extending a couple of layers into the film structure as well. Two distinct localized singly occupied states are created at about 0.7 eV above the VBM of the interface. These are the remaining unpaired electrons present in the first contact layer of CuO, left in the dangling bonds when the surface was cleaved from the bulk. They are clearly visible in the partial spin density shown on the right of Figure 3. Additionally, new empty hole states are found approximately 0.9 eV above the VBM and 0.8 eV below the continuous onset of empty conduction states. Those are located almost entirely on the Cu atoms at the Cu_2_O substrate side which formed a bond with O atoms from the film structure but remained coordinatively unsaturated and missing electrons to completely populate their d-shell.

Further away from the interface region, the bulk electronic gap of CuO obtained earlier is recovered before it reduces again when approaching the surface region of CuO(−111)_conv_, although, since CuO was strained to accommodate onto the lattice of Cu_2_O, its electronic structure has further altered. The recalculated gap of the strained CuO(−111)_conv_ is about 0.2 eV lower than the unstrained surface, which corresponds well to the behavior observed on the film side of the epitaxy between CuO and Cu_2_O.

This band alignment picture, obtained from the LPDOS, is significantly different from that obtained by assuming that the independent compounds are aligned without substantial changes to their electronic structure. First, the newly formed interface states are entirely missing in the model of independent compounds, as a result of the neglected bonding mechanism. Second, the respective VB and CB positions have moved with respect to each other and a substantially different alignment is created. This was additionally confirmed by the computed interface dipole and evaluated charge density difference. The formation of the CuO/Cu_2_O interface results in a net-zero interface dipole, with a magnitude of 4.9 D and orientation pointing from the substrate to the film. From the computed charge density difference, a charge accumulation is visible on the CuO side, with a consequent charge depletion on the Cu_2_O side (see Figure S6), confirming the charge transfer process occurring at the interface upon contact. As both mechanisms confirm, there is a charge polarization effect present at the CuO/Cu_2_O interface that prevents electrons migrating toward the CuO film side. From the outlined discussion, it is concluded that the vacuum alignment of the two outlined materials is an inadequate predictor of the interfacial electronic structure as it underestimates the complexity and plethora of effects present at the interface.

The computed interfacial electronic structure suggests a different interpretation of the photovoltaic operation of the interface. Upon illumination of the Cu_2_O substrate side, away from the interface, the photogenerated holes experience a thermodynamic drive toward the junction (an offset of about 0.25 eV), where they either get trapped at the defect levels or progress to the CuO side (Figure S7). The return of holes is at the same time hindered by a thermodynamic barrier (0.7 eV across the whole interface). Upon the CuO film side irradiation, there is no hindering process for the photogenerated electrons to recombine with the respective holes as both the VB and CB edges are positioned so that they create a barrier to spatial separation. Upon interface illumination, carriers get promoted from the defect-like occupied to the defect-like unoccupied states present at the interface. There is a spatial separation component involved, as the low energy adsorption near the interface is a charge transfer excitation from the film into the substrate and from there no thermodynamic force is present that would prevent recombination. However, since the energy required to promote the electrons from the defect-like unoccupied state into the conduction band is around the same value as the fundamental excitation energy, that process is probable as well. There the electron could be either spatially separated to the Cu_2_O side, as there is a favorable CB onset of 0.35 eV, or to the CuO side where the onset is around 0.65 eV. The excited holes would undergo the same process, although partially as there is only one favorable potential onset toward the film side of about 0.45 eV. However, now that part of the photogenerated carriers is found spatially on the same (film) side of the junction, recombination is again possible and only carriers from the substrate side would contribute to an effective output voltage.

It is also worth highlighting that these interface defect-like gap states are neither a result of lattice mismatch nor strain from the Cu–O bonds under thermal oxidation, as proposed in previous works.^106,107^ The surfaces used to model the alignment were pristine, without any introduced defects, while the interface itself created strong bonds and showed good structural compatibility resulting in a high adhesion energy. There are in fact two sources of these newly formed interface states, where half of them are above the VBM and the other half below the CBM in the otherwise pristine gap. The first cause is the reduced coordination number of a part of the CuO surface Cu^2+^ cations, and the second is the interface bonding-induced change in oxidation state of a part of the Cu_2_O topmost Cu^1+^ cations. Interestingly, when one of the two O atoms linearly coordinating the Cu^1+^ cation is removed, no defect states are formed as the Cu d shell stays fully populated (e.g., on the pristine Cu_2_O(111) slab). However, when an O is bonded to the 2-fold coordinated Cu^1+^ cation, new hole states are formed and a magnetic moment is induced consequently via the present unpaired spin. The same holds vice versa; when an O vacancy is introduced around the Cu^2+^ cation, it leaves dangling bonds behind in the still partially unoccupied d shell, reducing the spin-only magnetic moment but not quenching it entirely. Such behavior at the interface was so far only speculated upon^108^ while our work demonstrates the explicit presence and source of these intrinsic states. Furthermore, the interface induced partial Cu_2_O magnetization could further corroborate the interface-mediated ferromagnetism, yet additional investigations should follow to elucidate this effect.

**Figure 4:**
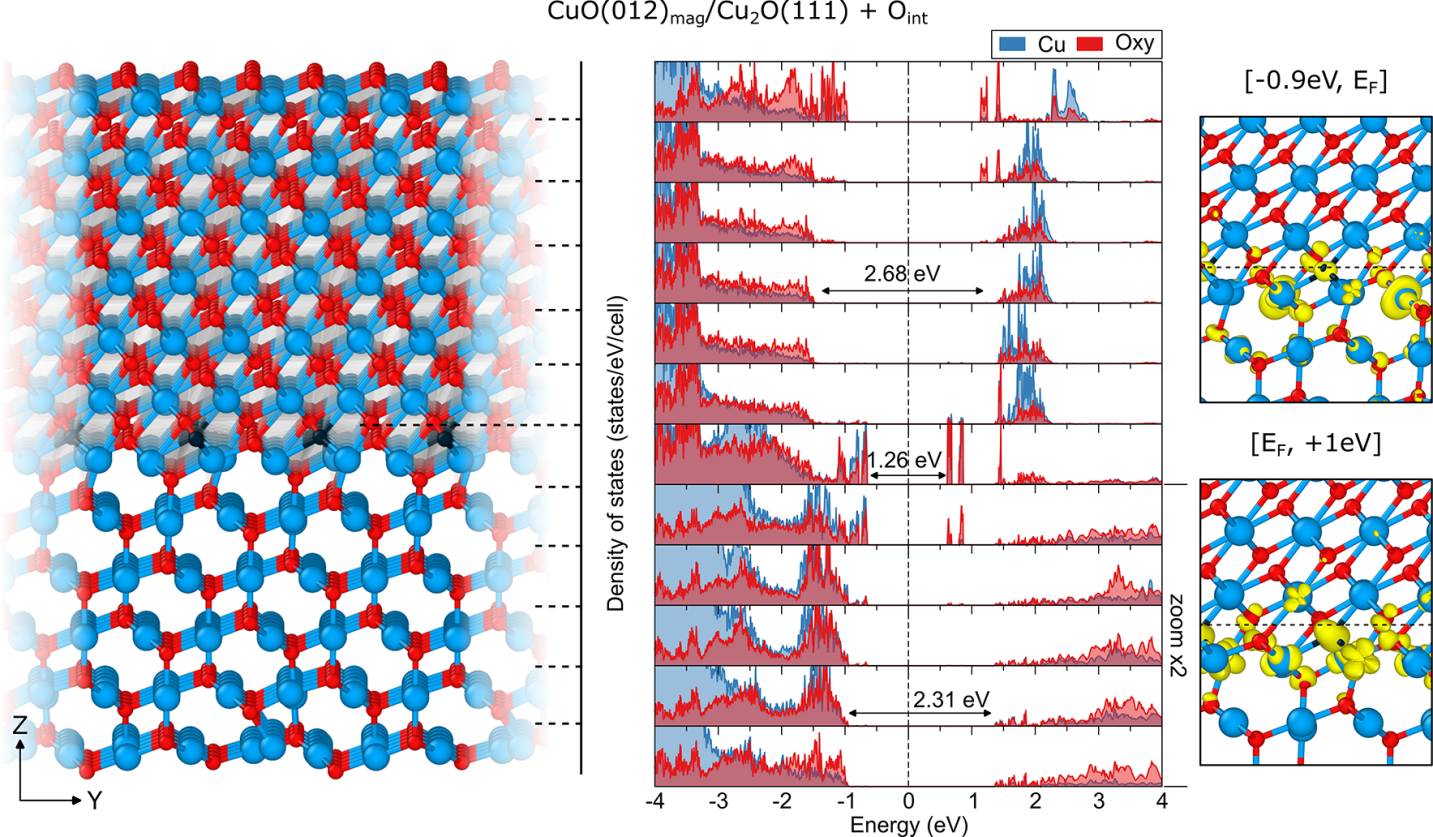
Relaxed atomic structure of the interface formed between the CuO(−111)_conv_ slab acting as a film and the Cu_2_O(111) slab acting as the substrate, with two interstitial oxygen atoms added per cell (left, interstitial oxygen colored dark blue) together with the species-resolved layer-projected electronic densities of states (LPDOS, middle) and partial spin-resolved densities around the Fermi level (right). Dashed lines in the relaxed structure indicate the layers separation chosen for the LPDOS projections. Zero on the energy axis of LPDOS refers to the Fermi level of the interface. Isosurfaces were reported for values of 0.005 e/Å^3^. Values were calculated using the B3LYP functional.

This situation begs the question of whether the undercoordinated Cu atoms could be saturated by a controlled oxidation process to effectively remove the dangling bonds, close the square planar arrangement, and clear the recombination states from the gap. In order to explore this possibility, we simulated the addition of interstitial oxygen at the interface layer, which would ideally mimic the experimental oxidation procedure as it is unlikely that one would be able to control the oxygen flow to an amount that would leave entirely pristine slabs, as in our first scenario. The relaxed LPDOS and relaxed structure of the CuO(−111)_conv_/Cu_2_O(111) interface with two additional oxygen interstitials per simulation cell are shown in Figure 4 (outlined alignment in Figure S8). Indeed, a positive gap alteration is achieved at the interface, with a new value of 1.26 eV, which is considerably larger than that of the nonoxidized one (0.89 eV). This is a result of the effective passivation of the CuO surface states and their merging with the VB of the Cu_2_O substrate. However, the improvement is only partial as the empty hole states below the CB are still present, now split by 0.20 eV and placed around 0.60 eV below the continuous CB states.

An additional difference between the pristine and defective interfaces is that the CuO film side is clear of states in the gap, while the fundamental electron transition upon irradiation in this scenario would take place entirely on the Cu_2_O substrate side. The defect-like hole states have shifted for an atomic layer to the substrate side and are still present 2–3 layers deep, similar to the case of the pristine interface. The source of this effect is clear: the added interstitial oxygen does form bonds with the 3-fold coordinated copper on the film side, but at the same time it also bonds with the otherwise 2-fold coordinated copper on the Cu_2_O side, propagating the process that occurred a layer above when the interface formed and part of the linearly coordinated Cu^1+^ atoms underwent a change in oxidation number. In other words, defect engineering at the CuO/Cu_2_O interface is effective up to a certain point after which it only serves to propagate the fundamental limitations of the interface along the contact region.

Although there is a clear increase in the band gap energy, detrimental interfacial charge-carrier recombination effects are not entirely suppressed and still affect the overall photovoltaic performance of the CuO/Cu_2_O heterostructure. A net-zero dipole across the interface is still present, with the same orientation as in the unoxidized interface (from substate to film) and a magnitude of 3.4 D that is reduced but entirely quenched to prevent interface polarization.

Albeit the calculations presented herein do not include an explicit description of electronic excitation and transport, they do reveal the fundamental limit of epitaxial processing of cuprous and cupric oxide. If one were able to achieve a perfect deposition of CuO on top of Cu_2_O (or vice versa, same results hold) without any impurities or defects, the geometric change in oxidation and coordination environment when going from Cu^2+^ to Cu^1+^ is always going to pose a hard limit on the maximal output performance of the operating device. Careful incorporation of defects, such as additional oxygen atoms in the lattice, does offer tunability of the defect-like states present at the interface and as such is potentially beneficial for the overall performance of the heterostructure.

## Conclusion

This work investigated the heterostructure epitaxy between CuO and Cu_2_O by means of hybrid density functional theory calculations. First, the computational soundness of the DFT setup was assessed by testing for the optimal basis set for the employed materials as well as suitable exchange–correlation functional and the bulk properties explored within. Bulk Cu_2_O properties are reproduced rather well, while the electronic properties of CuO are seemingly overestimated. However, a complex interplay between the geometry and electronic and magnetic degrees of freedom is elucidated in CuO, where the AFz magnetic ground state acts as a limit on the maximal band gap achievable in CuO.

Surface properties of Cu_2_O are found to converge to the respective bulk value rapidly, while this is not the case for CuO. The electronic gap varies considerably across different surfaces and spans a range of almost 1 eV, potentially explaining why experiments measure highly different values depending on the synthesis method and technique. Taking the relaxed slabs, first an independent compounds band alignment was created upon which the epitaxy between CuO and Cu_2_O was assessed. The experimentally noted alignment between the CuO(−111)_conv_ and Cu_2_O(111) surfaces is reproduced and an explicit heterostructure between a CuO film aligned over a Cu_2_O substrate modeled. The structure was relaxed and the band bending evaluated based on layer projected electronic densities of state. A non-negligible density of defects appears in the otherwise pristine electronic band gap at the interface. These defect-like states appear as a consequence of the reduced coordination environment of the Cu^2+^ cations present at the CuO side and from the induced charge transfer from the Cu^1+^ cation to the nearest binding O atom at the interface. Furthermore, controlled oxidation, calculated via the inclusion of oxygen interstitials at the interface, is proven to alter the gap substantially but does not overcome the underlaying limitations of the heterostructure entirely.

These structural defect-like interface states help explain the experienced underperformance the CuO/Cu_2_O interface shows, despite the postulated favorable band alignments as they promote charge recombination, which competes with the effective charge separation occurring at the interface structure.

## Supporting Information Available

Calculated lattice parameters and band gap energies of Cu_2_O and CuO with varying basis set parameters, exchange–correlation functional and amount of exact exchange; total-energy band gap convergence; spin fluctuations of the band gap of CuO; CuO relaxed surface energy dependence of the slab thickness; electronic structure of CuO surfaces; epitaxial matching between CuO acting as film and Cu_2_O acting as a substrate; charge density difference plots of the CuO/Cu_2_O interface with and without oxygen interstitials; band bending a the CuO/Cu_2_O interface (PDF)
