# Supplementary material for: Mind the Interface Gap: Exposing Hidden Interface Defects at the Epitaxial Heterostructure between CuO and Cu2O
Source: ACS Appl Mater Interfaces. 2022 Dec 8;14(50):56331–43. doi: 10.1021/acsami.2c16889 (PMC9782378; doi:10.1021/acsami.2c16889)
Supplement: Supplementary file 1 — am2c16889_si_001.pdf [file am2c16889_si_001.pdf]

Supplementary information

Mind the interface gap: exposing hidden interface defects at the epitaxial heterostructure between CuO and Cu_2O

Aleksandar Živković[1,2,], Giuseppe Mallia[2], Helen E. King[1], Nora H. de Leeuw[1], Nicholas M. Harrison[2]*

[1] *Department of Earth Sciences, Utrecht University, Princetonlaan 8a, 3584CB Utrecht, The Netherlands*
[2] *Department of Chemistry, Imperial College London, White City Campus, 80 Wood Lane, London W12 0BZ, United Kingdom*
**Corresponding author: a.zivkovic@uu.nl*

Table S1. DFT calculated (fully relaxed) lattice parameters and band gap energies of cubic Cu_2O for different combinations of exchange-correlation functionals and basis sets.

Functional	Van der Waals	Cu basis set	Oxy basis set	Cell parameter (Å)	E_g (eV)
Experiment				4.269[1]	2.17[2]
B3LYP	NaN	TZVPP-Linnera[3,4]	TZVPP-Linnera[3,4]	4.376	1.99
	D3	TZVPP-Linnera[3,4]	TZVPP-Linnera[3,4]	4.285	2.15
	D3	Towler/Harrison[5]	Valenzano2006[6]	4.069	3.33
	D3	Towler/Harrison[5] (added *d* shell)	Valenzano2006[6]	4.261	2.22
	D3	Doll2000[7]	Valenzano2006[6]	4.263	2.19
PBE0	NaN	TZVPP-Linnera[3,4]	TZVPP-Linnera[3,4]	4.316	2.35
	D3	TZVPP-Linnera[3,4]	TZVPP-Linnera[3,4]	4.265	2.44
HSE06	NaN	TZVPP-Linnera[3,4]	TZVPP-Linnera[3,4]	4.316	1.83
	D3	TZVPP-Linnera[3,4]	TZVPP-Linnera[3,4]	4.260	1.93
	D3	Towler/Harrison[5] (added *d* shell)	Valenzano2006[6]	4.237	1.97

Table S2. DFT calculated (fully relaxed) lattice parameters and band gap energies of monoclinic CuO for different combinations of exchange-correlation functionals and basis sets.

**Functional**	**Van der Waals**	**Cu basis set**	**Oxy basis set**	**Cell parameters (Å)**			**Angle**	**E_g (eV)**
				a	b	c	β(°)	
Exp[8]		(conventional cell)		4.72	3.40	5.04	99.5	1.3-1.5[9]
		(magnetic cell)		6.31	3.40	7.45	86.2	
B3LYP	NaN	TZVPP-Linnera[3,4]	TZVPP-Linnera[3,4]	6.573	4.137	6.829	77.8	2.41
	D3	TZVPP-Linnera[3,4]	TZVPP-Linnera[3,4]	6.312	3.396	7.537	84.9	3.05
	D3	Towler/Harrison[5] (added *d* shell)	Valenzano2006[6]	6.232	3.329	7.630	85.5	3.02
PBE0	NaN	TZVPP-Linnera[3,4]	TZVPP-Linnera[3,4]	6.404	3.451	7.471	84.7	3.84
	D3	TZVPP-Linnera[3,4]	TZVPP-Linnera[3,4]	6.247	3.310	7.597	85.7	3.98
HSE06	NaN	TZVPP-Linnera[3,4]	TZVPP-Linnera[3,4]	6.451	3.511	7.408	84.1	3.03
	D3	TZVPP-Linnera[3,4]	TZVPP-Linnera[3,4]	6.258	3.300	7.597	85.8	3.25
	D3	Towler/Harrison[5] (added *d* shell)	Valenzano2006[6]	6.151	3.242	7.697	86.3	3.19

Table S3. DFT single points calculated band gap energies of monoclinic CuO for different combinations of exchange-correlation functionals and basis sets.

**Functional**	**Cu basis set**	**Oxy basis set**	**Band gap (eV)**
B3LYP	TZVPP-Linnera[3,4]	TZVPP-Linnera[3,4]	2.99
	Towler/Harrison[5] (added *d* shell)	Valenzano2006[6]	2.89
	Doll2000[7]	Valenzano2006[6]	2.93
B1WC	Towler/Harrison[5](added *d* shell)	Valenzano2006[6]	2.34
WC1LYP	Towler/Harrison[5](added *d* shell)	Valenzano2006[6]	2.32
M06L	Towler/Harrison[5](added *d* shell)	Valenzano2006[6]	0.70
M06	Towler/Harrison[5](added *d* shell)	Valenzano2006[6]	3.33
SC-BLYP	Towler/Harrison[5](added *d* shell)	Valenzano2006[6]	2.47
LC-ωPBE	Towler/Harrison[5](added *d* shell)	Valenzano2006[6]	8.11
CAM-B3LYP	Towler/Harrison[5](added *d* shell)	Valenzano2006[6]	6.18
HISS	Towler/Harrison[5](added *d* shell)	Valenzano2006[6]	4.23
PBE0	Towler/Harrison[5](added *d* shell)	Valenzano2006[6]	3.88
HSE06	Towler/Harrison[5](added *d* shell)	Valenzano2006[6]	3.03

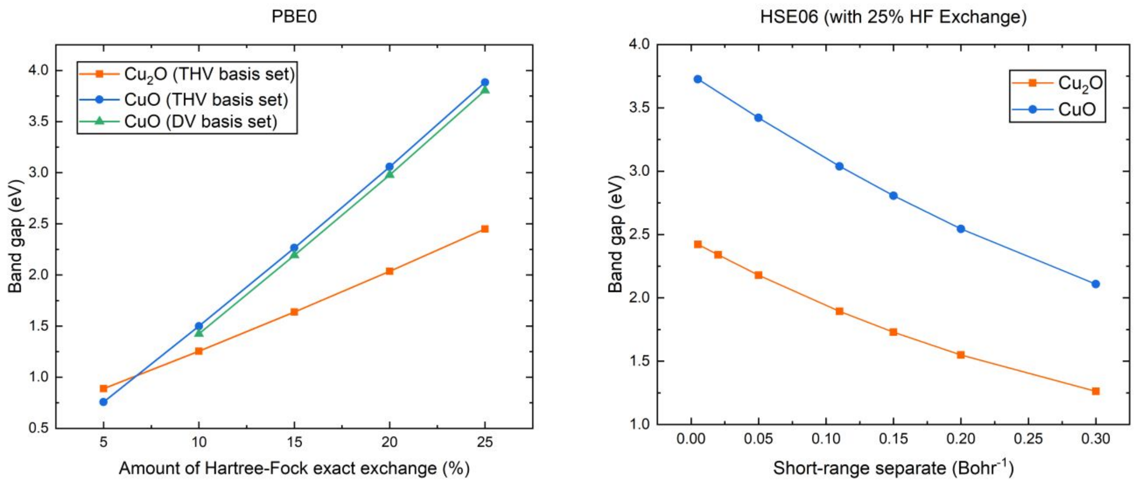

Figure S1. Tuning the amount of exact exchange entering the PBE0 functional (left) and the short-range separation parameter entering the HSE06 functional (right) for Cu_2O and CuO.

Table S4. Convergence of band gap energies estimated from total energy differences obtained using B3LYP with respect to the supercell size. No corrections were applied neither in the potential offset nor the interaction with the background charge.

Cu_2O		CuO	
Number of atoms (supercell size)	G (eV)	Number of atoms (supercell size)	G (eV)
6 (1x1x1)	3.16	16 (1x1x1)	2.87
48 (2x2x2)	2.57	92 (2x3x2)	2.96
162 (3x3x3)	2.40	720 (3x5x3)	2.88
384 (4x4x4)	2.33		
Bulk KS gap	2.22	Bulk KS gap	3.02

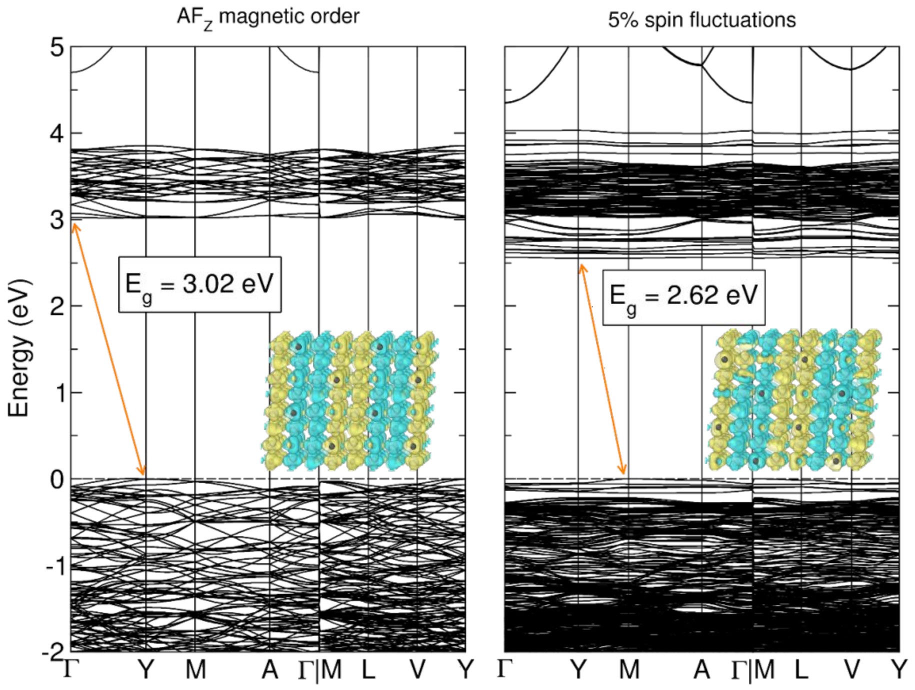

Figure S2. Influence of spin fluctuations on the bulk band gap of CuO. The inset shows the spin density for the corresponding simulation cells. Values obtained using B3LYP.

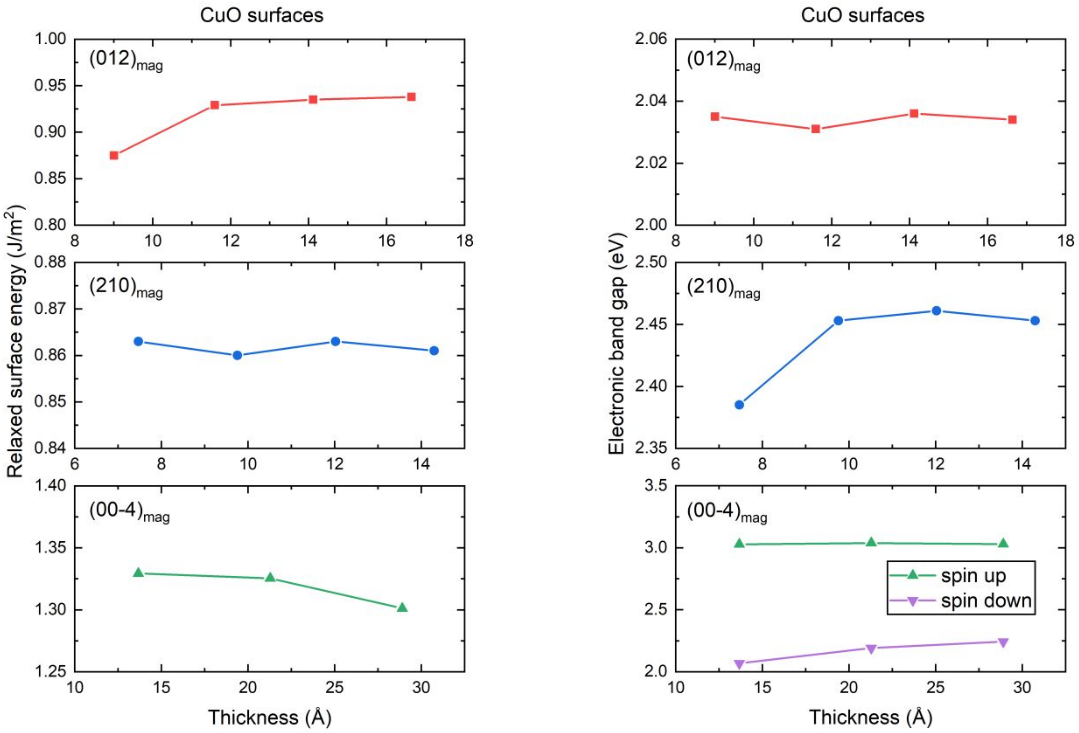

Figure S3. CuO relaxed surface energy (left) and electronic band gap (right) convergence with respect to increasing slab thickness. Values obtained using B3LYP.

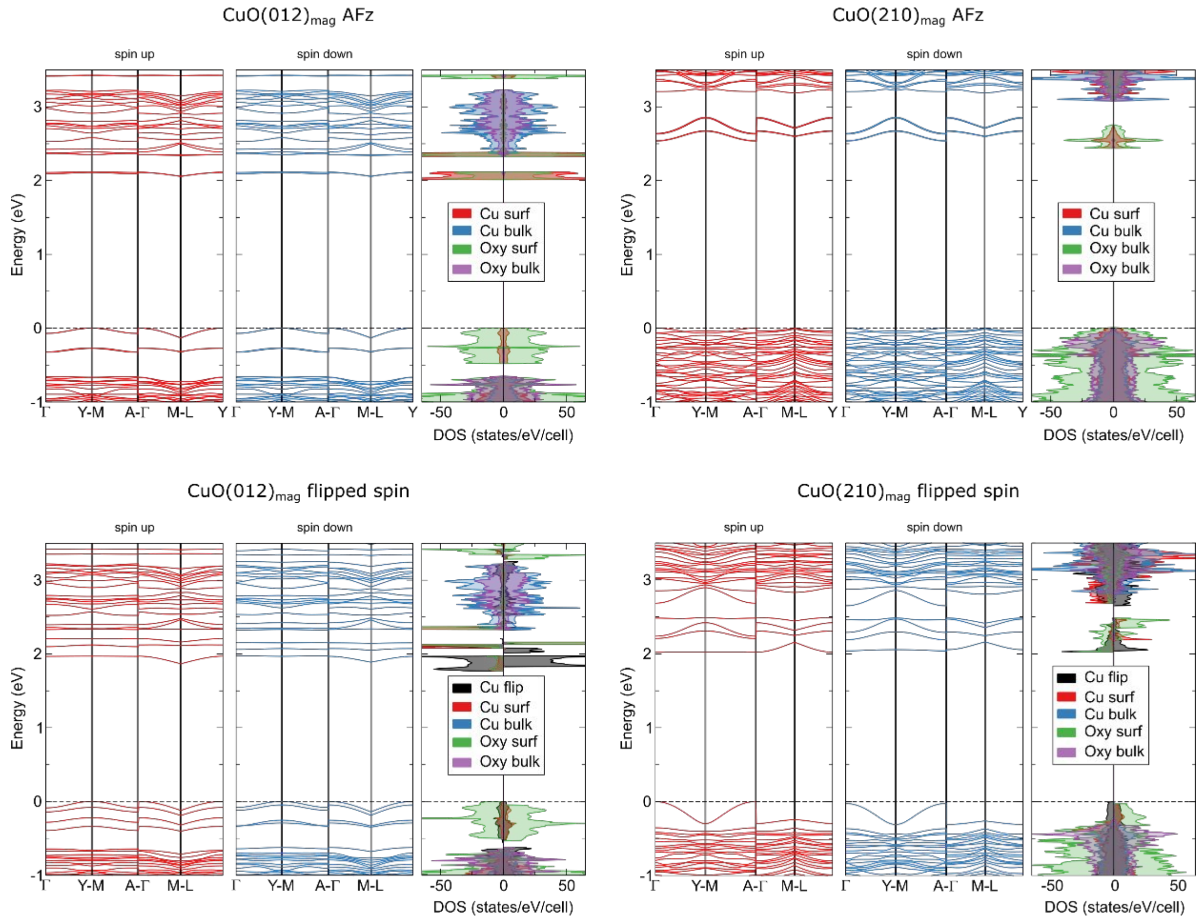

Figure S4. Two surfaces of CuO with their respective electronic band structure for the perfect spin arrangement in the ground state (AFz) and simulated spin flip structure. Results obtained using B3LYP.

Table S5. Corresponding notation of low surface Miller indices between the conventional and magnetic cell of CuO.

	Conventional cell	Magnetic cell
Miller index	(1 1 1)	(2 1 0)
	(-1 1 1)	(0 1 2)
	(0 1 1)	(1 1 1)
	(2 0 -2)	(0 0 -4)

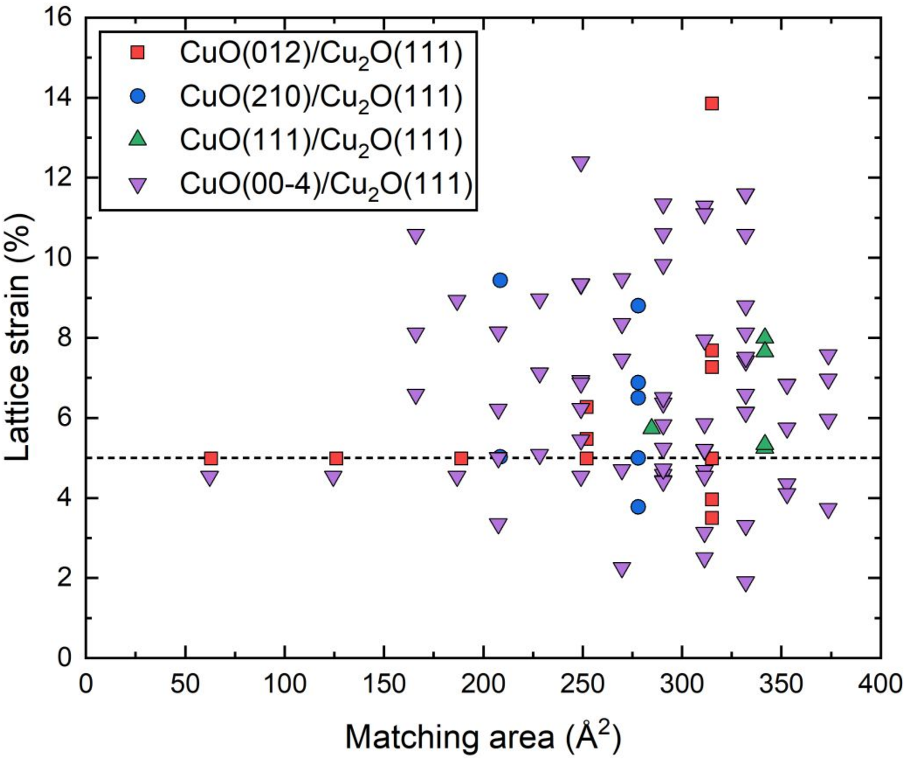

Figure S5. Epitaxial matching between CuO acting as film and Cu_2O acting as a substrate. Values obtained from bulk lattice parameters relaxed using the B3LYP functional.

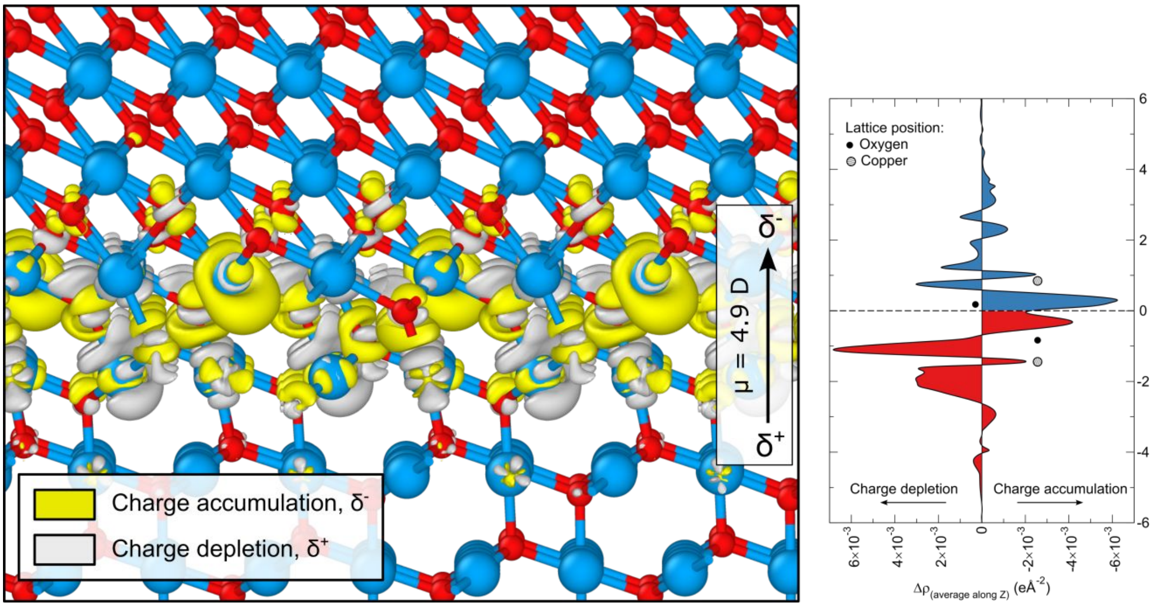

Figure S6. Charge density difference plot (left) of the $CuO(-111)_{conv}/Cu_2O(111)$ interface, where charge accumulation (yellow) and charge depletion (white) are shown, together with the plane averaged charge density difference (right). Isosurfaces reported for a value of 0.004 e/Å^3.

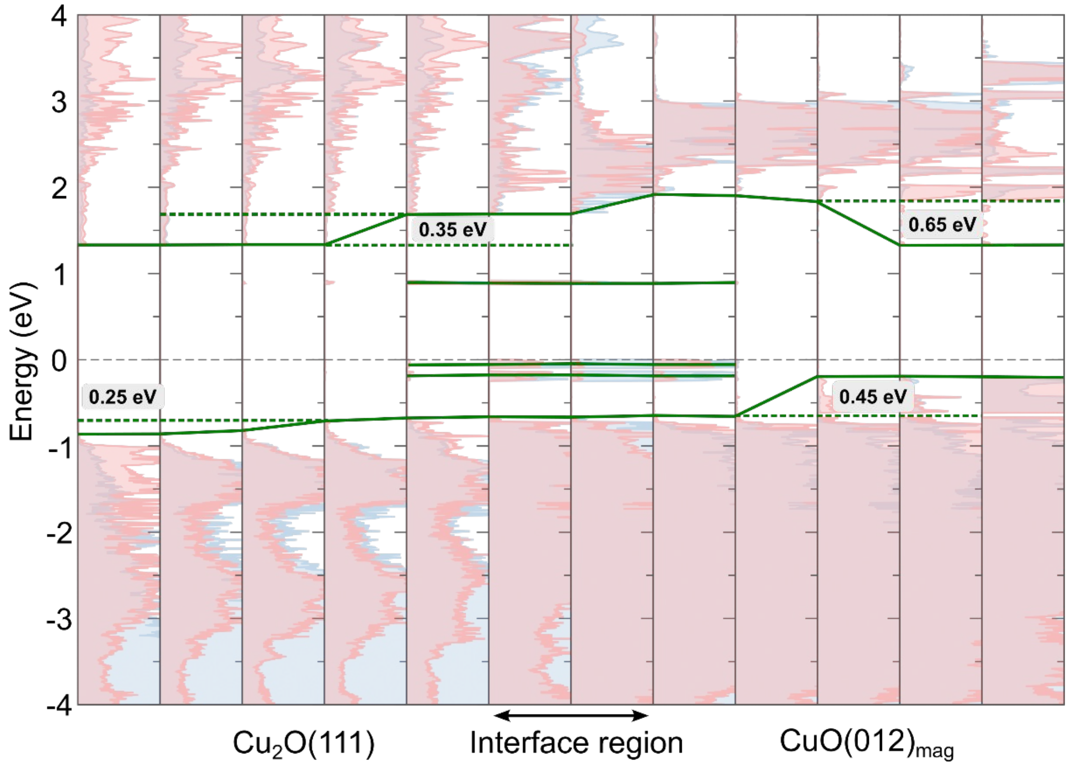

Figure S7. Outlining the band bending at the $CuO(012)_{mag}/Cu_2O(111)$ interface from the respective layer projected density of states. The full lines indicate obtained VBM and CBM positions, while the dashes lines serve a guide to the eye for a scenario where the defect states would be removed and the surface states present at the CuO side passivated, leaving a defect-free band structure.

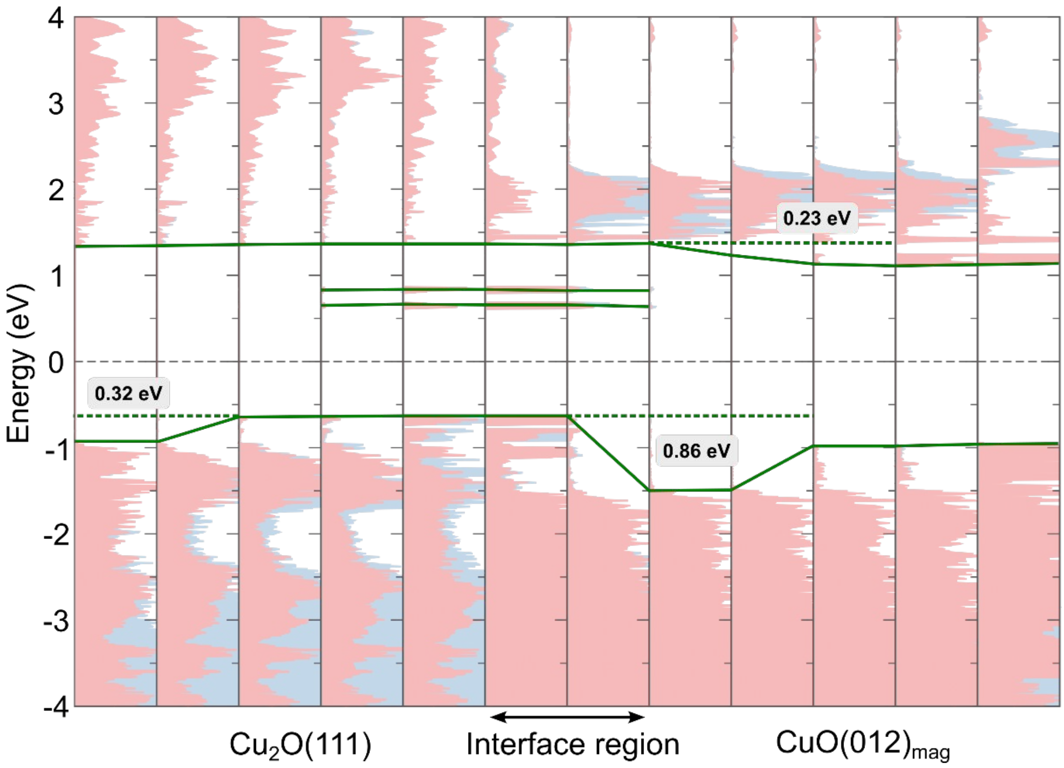

Figure S8. Outlining the band bending at the CuO(012)mag/Cu2O(111) + O_{int} interface from the respective layer projected density of states. The full lines indicate obtained VBM and CBM positions, while the dashes lines serve a guide to the eye for a scenario where the defect states would be removed and the surface states present at the CuO side passivated, leaving a defect-free band structure.

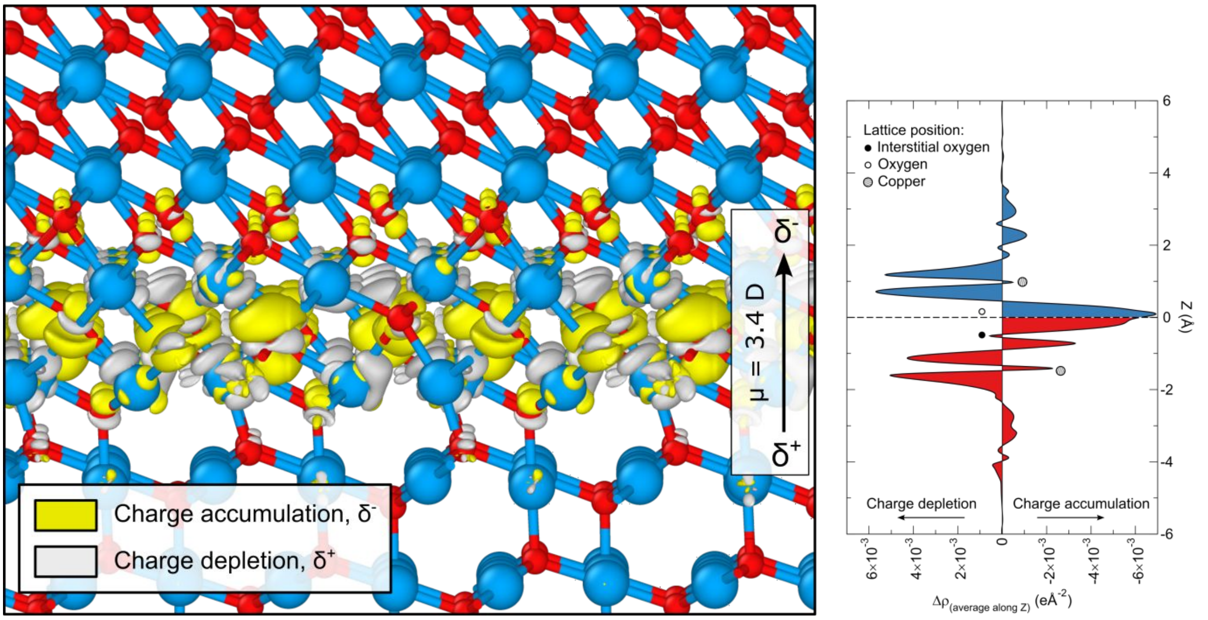

Figure S9. Charge density difference plot (left) of the CuO(-111)$_{conv}$/Cu_2O(111) + O_{int} interface, where charge accumulation (yellow) and charge depletion (white) are shown, together with the plane averaged charge density difference (right). Isosurfaces reported for a value of 0.004 e/Å^3.

References

(1) Werner, A.; Hochheimer, H. D. High-Pressure x-Ray Study of Cu_2O and Ag_2O. *Phys. Rev. B* **1982**, *25* (9), 5929–5934. https://doi.org/10.1103/PhysRevB.25.5929.

(2) Hu, J. P.; Payne, D. J.; Egdell, R. G.; Glans, P.-A.; Learmonth, T.; Smith, K. E.; Guo, J.; Harrison, N. M. On-Site Interband Excitations in Resonant Inelastic x-Ray Scattering from Cu_2O. *Phys. Rev. B* **2008**, *77* (15), 155115. https://doi.org/10.1103/PhysRevB.77.155115.

(3) Linnera, J.; Karttunen, A. J. Ab Initio Study of the Lattice Thermal Conductivity of Cu_2O Using the Generalized Gradient Approximation and Hybrid Density Functional Methods. *Phys. Rev. B* **2017**, *96* (1), 014304. https://doi.org/10.1103/PhysRevB.96.014304.

(4) Linnera, J.; Sansone, G.; Maschio, L.; Karttunen, A. J. Thermoelectric Properties of P-Type Cu 2 O, CuO, and NiO from Hybrid Density Functional Theory. *J. Phys. Chem. C* **2018**, *122* (27), 15180–15189. https://doi.org/10.1021/acs.jpcc.8b04281.

(5) Towler, M. D.; Dovesi, R.; Saunders, V. R. Magnetic Interactions and the Cooperative Jahn-Teller Effect in ${\mathrm{KCuF}}_{3}$. *Phys. Rev. B* **1995**, *52* (14), 10150–10159. https://doi.org/10.1103/PhysRevB.52.10150.

(6) Valenzano, L.; Torres, F. J.; Doll, K.; Pascale, F.; Zicovich-Wilson, C. M.; Dovesi, R. Ab Initio Study of the Vibrational Spectrum and Related Properties of Crystalline Compounds; the Case of CaCO3 Calcite. *Zeitschrift fur Phys. Chemie* **2006**, *220* (7), 893–912. https://doi.org/10.1524/zpch.2006.220.7.893.

(7) Doll, K.; Harrison, N. . Chlorine Adsorption on the Cu(111) Surface. *Chem. Phys. Lett.* **2000**, *317* (3–5), 282–289. https://doi.org/10.1016/S0009-2614(99)01362-7.

(8) Samokhvalov, A. A.; Viglin, N. A.; Gizhevskij, B. A.; Loshkareva, N. N.; Osipov, V. V.; Solin, N. I.; Sukhorukov, Y. P. Low-Mobility Charge Carriers in CuO. *Zhurnal Eksp. i Teor. Fiz. (ISSN 0044-4510)* **1993**, *103* (April 1992), 951–961.

(9) Marabelli, F.; Parravicini, G. B.; Salghetti-Drioli, F. Optical Gap of CuO. *Phys. Rev. B* **1995**, *52* (3), 1433–1436. https://doi.org/10.1103/PhysRevB.52.1433.
